# Narcissism between cold-blooded and hot-headed characters in toxic online behaviors. The moderating role of the R^4^ functional drivers

**DOI:** 10.3389/fpsyg.2025.1562635

**Published:** 2025-05-30

**Authors:** Oronzo Mazzeo, Giuseppe La Selva, Manuela Longo, Lucia Monacis

**Affiliations:** ^1^Department for the Promotion of Human Science and Quality of Life, San Raffaele Telematic University, Rome, Italy; ^2^Department of Humanities, University of Foggia, Foggia, Italy

**Keywords:** narcissism, grandiose, vulnerable, functional driver, cyberbullying, trolling, toxic, online behavior

## Abstract

**Introduction:**

Previous studies on the association of narcissism with toxic online behaviors have shown mixed results, ranging from a weak relationship with cyberbullying to no relationship with trolling behavior. Moreover, there has been no clear distinction on which specific dimension (grandiose or vulnerable) is related to online aggressive behaviors. Therefore, the present study examined the relationships of the two variants of narcissism with cyberbullying and trolling behavior, taking into account the moderating role of four different functional drivers.

**Method:**

Three self-reported questionnaires were administered to 202 undergraduate students. The survey included a sociodemographic section, the grandiose and vulnerable Narcissism dimension, the tendency towards engaging in cyberbullying and/or trolling behaviors, and four functional drivers of cyber-aggression.

**Results:**

Findings indicated individual differences between the two variants of narcissism associated with the two cyber-aggressive behaviors. Correlational analyses showed that (i) vulnerable narcissism was consistently related to cyberbullying and trolling behavior; (ii) grandiose narcissism was neither related to cyberbullying nor to trolling behavior. Moderation analyses indicated that (iii) grandiose narcissists were more likely to show trolling behavior if they exhibited high levels of functional driver (reward and revenge), and were less likely to be engaged in cyberbullying behavior if they displayed lower levels of revenge; (iv) vulnerable narcissists were more likely to show trolling behavior if they manifest high levels of revenge. Finally, the findings reported no other moderated effects of the motivational drivers on cyberbullying behavior for vulnerable narcissists.

**Discussion:**

This study provided evidence for the dual behavioral mode of the variants of narcissism in cyber-aggression, thus discovering the antagonistic aspect underlying both variants of the trait in connection with the four functional drivers. Further studies should not only confirm such empirical evidence, but also develop effective moderation intervention tailored to a more detailed users’ personality profile for reducing trolling and cyberbullying behaviors.

## 1 Introduction

The widespread use of the Internet through social media and discussion groups has triggered an increasing proliferation of a range of toxic online behaviors, such as cyberbullying and trolling. The former is defined as “any behaviors performed through electronic media by individuals or groups of individuals that repeatedly communicate hostile or aggressive messages, intended to inflict harm or discomfort on others” (Tokunaga, 2010). The latter is defined as a specific type of malicious online behavior, intended to aggravate, annoy, or otherwise disrupt online interactions and communication (Bishop, 2012). Although both offensive behaviors entail the use of aggressive online tactics, their targets are different: trolling is employed to create emotional distress among strangers, whereas cyberbullying is directed towards individuals with whom the perpetrator has a personal connection (Hardaker, 2010). The rise of malicious dark participation has led researchers to reduce its severe effects by implementing effective counter-strategies (Quandt et al., 2022) following a data-driven-based approach which is focused on machine learning-based solutions: by using behavioral data, the personality profiling of the users (perpetrators) has been inferred through the textual and semantic analysis of social media posts on platforms (Bilewicz et al., 2021; Hangartner et al., 2021; Trujillo and Cresci, 2023). Although such studies provided important insights on novel patterns not predicted by existing theories, they generally lacked conceptual precision, psychometric evaluation, and nomological assessment, probably due to the tendency to exclude personality science experts (Phan and Rauthmann, 2021). On the contrary, following a theory-based approach focused on the personality perspective, other researchers have investigated personality characteristics by examining the associations of the dark side of personality traits with two toxic online behaviors. In particular, Moor and Anderson (2019) showed that Narcissism was the trait least consistently related to antisocial online behaviors: compared to other dark traits (psychopathy, Machiavellianism and everyday sadism), it resulted to be weakly related to cyberbullying and unrelated to trolling behavior. However, a recent meta-analysis on relationships between dark triad traits and cyberbullying (Xu et al., 2024) has reported moderate positive correlations of cyberbullying with the three traits (narcissism, psychopathy and Machiavellianism). Therefore, there is no consensus on the relationship between narcissism and online offensive behaviors.

It should also be noticed that a large proportion of relevant studies did not distinguish between different types of narcissism in offensive behaviors on social media. Although a structural model of narcissism was formulated, where the juxtaposition of two independent dimensions—grandiose and vulnerable narcissism—has been empirically confirmed (Miller et al., 2011), Kjærvik and Bushman (2021) have focused on the connections between narcissism and aggression and pointed out that (i) just few investigations (*n* = 9) found a positive relationship between narcissism and trolling behavior, (ii) narcissism was strongly positively related to any forms of aggression, with a stronger relationship to offline than online bullying, and (iii) both aspects of the trait were generally related to aggression—even if no clear distinction emerged regarding the specific dimension linked to aggressive behaviors.

Recently, a couple of studies (Chen et al., 2022; Rohmann et al., 2024) has started to explore the two facets of narcissism in connection with aggression and trolling behavior and showed individual differences between the two dispositional aspects in aggressive tendencies online.

Worthy of note is the question of what psychological mechanisms underlie narcissistic actions or, put differently, which principal motives—linked to functions of aggression associated with the grandiose and vulnerable dimensions—could moderate the two toxic online behaviors. In order to answer such a research question, it could be useful to combine the two main models concerning the aggressive inclinations of narcissism, the threatened egotism (Baumeister et al., 1996) and the Status Pursuit in Narcissism (SPIN) (Grapsas et al., 2020) with the recent quadripartite model of the function of cyber-aggression (Runions et al., 2017). Exploring this trajectory of research could provide further insights into the question, because it may help identify particular intervention strategies to address risk factors that increase the likelihood of narcissistic individuals to be engaged in cyber-aggressive behaviors.

### 1.1 The two dimensions of narcissism and their associations with aggression

An increasing number of scholars agree that everyday narcissism is not a singular construct, although not all researchers share a common nomenclature of its specific dimensions. The Narcissism Spectrum Model (NSM) (Krizan and Herlache, 2018), which identifies entitlement as the core element, and grandiosity and vulnerability as the two peripheral components, provides a comprehensive framework for understanding individual differences in narcissistic personality on the basis of two separate functional orientations, namely boldness and reactivity. Since the two orientations reflect different personality-environment transactions, determined by specific aspects of temperament and reinforced by matching self-regulation styles, they underlie the psychosocial features of narcissistic grandiosity and vulnerability at both personality and social–behavioral levels of analysis.

The first orientation, boldness, is described as a heightened motivational orientation toward seeking rewarding experiences, often raising concerns about risks or costs associated with reward pursuit (Block and Block, 1980). Its related concepts, including fearless dominance, daringness, and eagerness (Patrick et al., 2009), share strong appetitive and exploratory tendencies of the narcissistic grandiosity. Individuals with high levels of grandiose narcissism display self-confidence in their own abilities, coupled with feelings of grandiosity and a tendency to seek attention and admiration from others. Being characterized by traits associated with aggression and dominance (Miller and Maples, 2011), they are also referred to as “hard-headed, aggressive, and a show-off” (Wink, 1991). The second orientation, reactivity, indicates a stress-prone and volatile disposition dominated by high avoidance motivation and characterized by an attitude towards detecting and combating threats to self-image. Concepts related to reactivity include anxiety and emotional dysregulation (Ruocco et al., 2013), both of which share strong aversive and avoidance tendencies that interfere with approach goals. Narcissistic vulnerability is built on a reactive orientation and focuses on avoidance and ‘fight-flight’ responses. Individuals with a high level of vulnerable narcissism are characterized by egocentrism, hypersensitivity to evaluations from others, defensiveness, anxiety, self-indulgence, conceitedness, arrogance, and an insistence on prevailing over others (Wink, 1991).

To explain the psychological mechanisms underlying narcissistic aggression, researchers developed two models based on the desire to have a positive self-image. The first, the threatened egotism model, which is linked to intrapersonal motive, postulates that narcissists are likely to retaliate against those who evaluate them negatively, owing to their inflated egos and their ‘think skins’ (fragile egos); the second, the SPIN model, which is linked to interpersonal purposes, assumes that narcissists are prone to be aggressive against others in their attempts to obtain a dominant status and social benefits and to persuade others of their superior skills and abilities. Therefore, being consistent with Grapsas et al. (2020), the SPIN model explains the self-regulation process of the grandiose narcissism, whereas the threatened egotism model is related to the aggression expressed by the vulnerable facet of the trait. Furthermore, the two models can be theoretically related to the classical distinction between the two functions of aggressive behavior, reactive vs. proactive (Bushman and Anderson, 2001). In fact, individuals with high levels of vulnerable narcissistic traits, characterized by fragile egos and a style of reactivity orientation in their personality–environment transactions, tend to show reactive aggression (also called hostile, emotional, affective, and impulsive aggression), also known as a “hot tempered” annoyance-based aggression, which is an “end in itself.” On the other hand, individuals exhibiting high levels of grandiose narcissism, driven by seeking attention and admiration from others in order to obtain a social status, tend to manifest proactive aggression (also called instrumental and premeditated aggression), referred to as “cold-blooded” incentive-based aggression that is a “means to some other end” (e.g., money, status, power, reputation, revenge, prestige).

### 1.2 The four functional drivers in cyber-aggression

A further step forward in this area involves shifting from the classical distinction between reactive and proactive aggression to an emphasis on motivations/functions in relation to the specific context-related properties, in which aggressive behaviors appear. Consistent with the general aggression model, which assumes that situational factors influence aggression by influencing cognition, affect, and arousal (Anderson and Bushman, 2002), aggressive behavior in online environments can exhibit a heterogeneity of why (functional drivers). Accordingly, the two aggressive models can be integrated with the quadripartite model of the functions in cyber-aggression (Runions, 2013)—henceforth referred to as the R^4^ model of cyber-aggression. Following the quadripartite violence typology (QVT; Howard, 2011), the R^4^ model is based on the intersection of the two orthogonal dimensions, affective motive or motivational valence (appetitive vs. aversive) and self-regulatory control (impulsive vs. controlled) applied in the context of cyber-aggression. The nomenclature of each functional driver/motivation was also simplified with an alternative wording: impulsive-aversive aggression as “rage aggression,” controlled-aversive aggression as “revenge aggression,” controlled appetitive aggression as “reward aggression,” and impulsive appetitive aggression as “recreation aggression” (Runions et al., 2017). The rage aggression and revenge aggression are referred to traditionally operationalized forms of reactive aggression, since aggression is driven by intrapersonal motives and displayed as a response aimed at the removal of negative emotional states. It is conducted in an impulsive or controlled manner, respectively. The remaining two functions, reward and recreation aggression, which map onto traditional conceptualizations of proactive aggression, are driven by interpersonal motives as responses in the pursuit of some environmental rewards or appetitive gains, and are conducted in a controlled or impulsive manner, respectively.

To the authors’ knowledge, no study has examined the underlying motives behind toxic online behaviors by disentangling individual differences in narcissistic, aggressive online actions. The emerging results could spur new directions in cyber-aggression research by considering how affective (appetitive vs. aversive) and self-control (impulsive vs. controlled) processes in individuals with high levels of vulnerable and grandiose narcissism can motivate acts of online aggression and alter the expression of such aggressive behaviors. The analysis of the moderating role of the different functional drivers that condition the paths from the two variants of narcissism to cyber-aggressive behaviors could be useful to plan effective interventions tailored to users’ personality profile. In light of such premises, it was predicted that:

- drawing from existing findings (Kjærvik and Bushman, 2021; Xu et al., 2024) and in line with the theoretical frameworks regarding the aggressive inclinations of narcissism (the threatened egotism model and the SPIN model), both facets of narcissism (grandiose and vulnerable) will be associated with cyberbullying (H1 Model 1 and H1 Model 2) and trolling behavior (H2 Model 1 and H2 Model 2);
- cyber-aggressive behaviors will be the result of a function of interaction effects of the different functional drivers with the two narcissistic variants.

More specifically,

- following the SPIN hypothesis, the association between grandiose narcissism and the two toxic online behaviors will be strengthened by high levels of controlled functional drivers (H3 Model 1 and H4 Model 1);
- following the threatened egotism hypothesis, the association between vulnerable narcissism and the two toxic online behaviors will be strengthened by high levels of aversive functional drivers (H3 Model 2 and H4 Model 2).

## 2 Method

### 2.1 Procedure and participants

The study was carried out in accordance with the European Code of Conduct for Research Integrity (ECCRI) on the conduct of experiments involving human participants. The researchers adhered to the ethical standards outlined in the Ethics Code of the Italian Association of Psychology (AIP) when conducting this investigation. Additionally, the university’s local ethics committee approved the survey protocol (with the assigned code number: 17/2024). The anonymity of the data was ensured and no personal information was collected from the participants. Each participant provided their informed consent to take part in the investigation on a voluntary basis; they could withdraw from the survey at any time for any reason.

Before data collection, an *a priori* power analysis was performed using G*Power (Buchner et al., 2024) to determine the optimal sample size. The analysis indicated that a minimum of 199 participants were required for moderation analyses based on multiple linear regression to demonstrate an effect size of 0.02 with a power of 0.80 and alpha level = 0.05. Accordingly, the sample size was 202 participants.

The participants were undergraduate students. The initial sample comprised 388 subjects. Following the application of a data cleaning procedure, 186 subjects were excluded due to incomplete self-report data or the repeated completion of the same self-report. Moreover, identical responses, if provided, were detected through straight-liners across all scale questions. The final research sample consisted of 202 subjects (MAge = 23.00, SD = 6.67; Males = 88, Females = 114; MAge males = 23.70, SD = 6.53; MAge females = 22.54, SD = 6.76). Data was collected using a battery including three self-report instruments and was implemented via an electronic survey format using the LimeSurvey server (LimeSurvey GmbH, 2024).

### 2.2 Measures

#### 2.2.1 Narcissism

Both dimensions of the narcissistic trait were assessed with the subscales of the Grandiose Entitlement and the Entitlement Rage belonging to the Dark Side of Humanity Scale (Katz et al., 2022). The two subscales include 16 statements comprising nine items for Grandiose Entitlement and seven items for Entitlement Rage. Participants were invited to indicate their level of agreement rated on a 6-point Likert scale, ranging from “Not at all like me” to “Very much like me.” A high score on both subscales indicates high levels of grandiose and/or vulnerable narcissism, respectively. In the current study, the internal consistency for both aspects was adequate: alpha and omega values ranging from 0.76 and 0.75 for Grandiose Entitlement to 0.84 for the Entitlement Rage dimension. They were slightly lower than those reported by Katz et al. (2022).

#### 2.2.2 Motivational valence and self-regulatory control

The Cyber-Aggression Typology Questionnaire (CATQ; Runions et al., 2017) was administered to capture the diversity of function of cyber-aggression. The scale comprises 29 items rated on a 4-point Likert scale, ranging from “Not at all true of me” to “Very true of me.” Some examples of the item for each function are “I overreact before I have a chance to think about the consequences when someone says something mean online” (impulsive-appetitive), “I get back at people who make fun of me on the Internet because their posts hurt more the more I think about them” (controlled-appetitive), “Sometimes I will team up with my friends to bring someone down online” (impulsive-aversive), and “I make fun of people I do not know on the internet without thinking about whether they might be will see hurt it” (controlled-aversive). A high score in the impulsive-aversive or controlled-aversive quadrants indicates a tendency towards reactive aggressive behavior, respectively, without or with self-control capacity. Similarly, high scores in the impulsive-appetitive or controlled-appetitive quadrants indicate a tendency to act aggressively in pursuit of an environmental and personal reward, respectively, without or with self-control capacity. The Cronbach’s alpha and McDonald’s omega coefficients for each score range from 0.798 to 0.891, thus indicating a good internal consistency of the instrument.

#### 2.2.3 Cyberbullying and trolling behavior

The tendencies towards engaging in cyberbullying and/or trolling behaviors were assessed with the 27-item Cyberbully/Troll Deviancy Scale (CTDS; Zezulka and Seigfried-Spellar, 2016). The instrument includes 14 items related to various cyberbullying behaviors and 13 items related to trolling behaviors. Using a five-point Likert scale, the subjects indicated the frequency with which they had engaged in online activities with known individuals (cyberbullying) or with unknown individuals (trolling) over the past five years. The reliability of CTDS ranged from 0.703 to 0.862 for values of alpha and omega coefficients, respectively.

### 2.3 Analysis strategy

**Figure 1:**
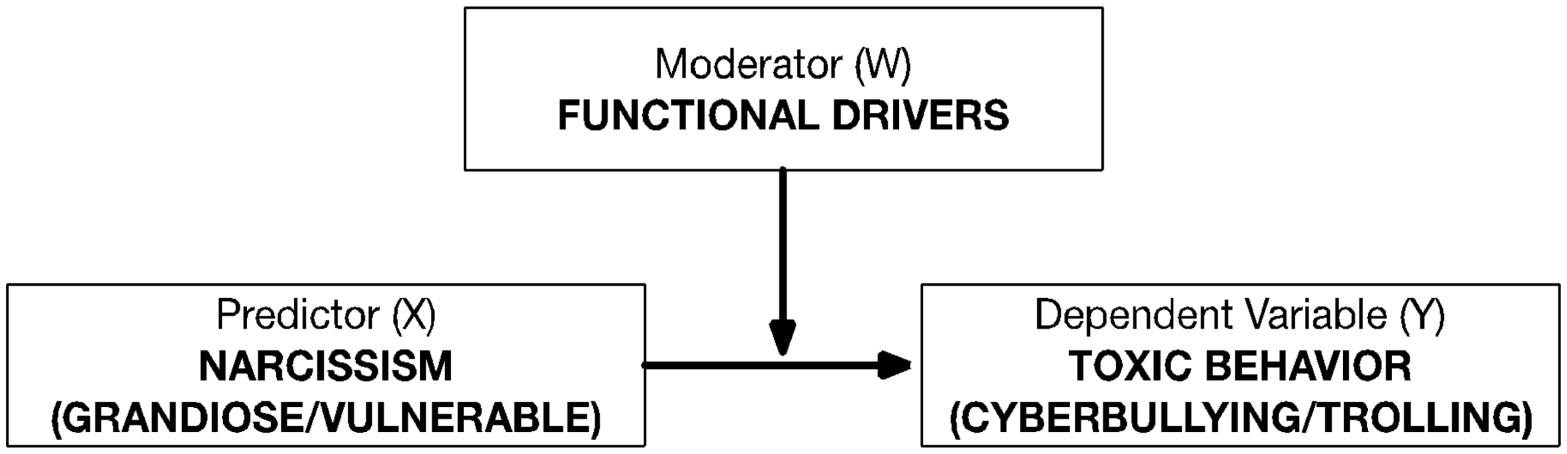
Conceptual representation of the moderation model for hypotheses H3 and H4.

A cross-sectional design was employed to test the hypotheses. Before testing the hypothesized associations, the instruments were translated into Italian—using the forward and backward method—by two independent native English professionals with previous experience in psychology research. The two translated texts were subsequently merged into a single document and reviewed against the original text to identify any discrepancies in meaning. No inconsistencies were singled out. To test the hypothesised associations (H1 and H2), bivariate and partial correlations were conducted taking into account two models for each narcissism trait (Model 1 and Model 2). Furthermore, the moderating role of the different functions of cyber-aggression was tested by considering the relationship between narcissistic traits and the two toxic online behaviors (H3 and H4). The conceptual representation of the moderation model is portrayed in Figure 1. Similar to the previous hypotheses, the two models for each narcissistic trait (Model 1 and Model 2) were examined with a specific functional driver, employed as the moderator, for each online aggressive behavior that was considered as the dependent variable. Regarding the data analysis strategy, authors applied the simple slopes procedure using the robust statistical tool Hayes PROCESS Macro (Hayes, 2022) with bootstrapping. Additionally, False Discovery Rate (FDR) correction was employed to address potential false positives due to the accumulation of alpha error and adjust significance thresholds.

## 3 Results

3.1. The means and standard deviations of all measures of interest in the total sample, in males and females, are presented in Table 1. Moreover, results of t-test with their associated 95% CIs, and each effect sizes are also reported.

**Table 1:** Descriptive statistics of all variables of interest.

Variable	Total mean	SD	Male mean	SD	Female mean	SD	t (*p*)	95% CI	Cohen‘s d
Grandiose narcissism	2.006	0.722	1.950	0.787	2.050	0.668	0.956	(−0.143, 0.414)	0.136
Vulnerable narcissism	2.639	1.045	2.540	0.988	2.720	1.080	1.240	(−0.103, 0.4545)	0.176
Cyberbullying	1.139	0.236	1.160	0.269	1.120	0.206	−1.317	(−0.465, 0.092)	−0.187
Trolling	1.106	0.309	1.190	0.442	1.040	0.101	−3.505^***^	(−0.779, −0.2144)	−0.497
Recreation	1.262	0.352	1.260	0.352	1.260	0.354	−0.119	(−0.295, 0.2613)	−0.017
Reward	1.136	0.365	1.160	0.431	1.110	0.305	−0.944	(−0.412, 0.1447)	−0.134
Rage	1.075	0.284	1.110	0.372	1.050	0.188	−1.402	(−0.477, 0.0802)	−0.199
Revenge	1.059	0.233	1.070	0.256	1.050	0.214	−0.592	(−0.362, 0.1944)	−0.084

Significance: ^***^p < 0.001.

The supposed associations of the two traits with cyberbullying (H1 Model 1 and H1 Model 2) and trolling behavior (H2 Model 1 and H2 Model 2) were tested using bivariate and partial correlations (Table 2). Regarding the association between grandiose narcissism and cyberbullying (H1 Model 1), the findings were inconsistent, showing a significant association in bivariate correlation and no association in partial correlation. On the contrary, a significant association between vulnerable narcissism and cyberbullying (H1 Model 2) emerged in both bivariate and partial correlations. Furthermore, while the supposed relationship between grandiose narcissism and trolling behavior (H2 Model 1) was not confirmed in bivariate and partial correlations, the link between vulnerable narcissism and trolling behavior (H2 Model 2) was confirmed in both analyses.

**Table 2:** Bivariate and partial correlations among variables of interest.

Variable	DSHS-ER	Cyberbullying	Trolling	Recreation	Reward	Rage	Revenge
DSHS-GE	0.614^**^	0.185^**^ (−0.030)	0.102 (−0.007)	0.348^**^	0.314^**^	0.292^**^	0.257^**^
DSHS-ER	-	0.338^**^ (0.289^***^)	0.175^*^ (0.143^*^)	0.488^**^	0.376^**^	0.244^**^	0.229^**^
Cyberbullying	-	-	0.516^**^	0.471^**^	0.420^**^	0.382^**^	0.350^**^
Trolling	-	-	-	0.430^**^	0.506^**^	0.566^**^	0.474^**^
Recreation	-	-	-	-	0.686^**^	0.537^**^	0.538^**^
Reward	-	-	-	-	-	0.823^**^	0.780^**^
Rage	-	-	-	-	-	-	0.849^**^

^*^p < 0.05; ^**^p < 0.01; ^***^p < 0.001. Partial correlations in parentheses controlling for confounding variable (grandiose or vulnerable); DSHS-GE = Grandiose Entitlement (Grandiose Narcissism); DSHS-ER = Entitlement Rage (Vulnerable Narcissism).

**Figure 2:**
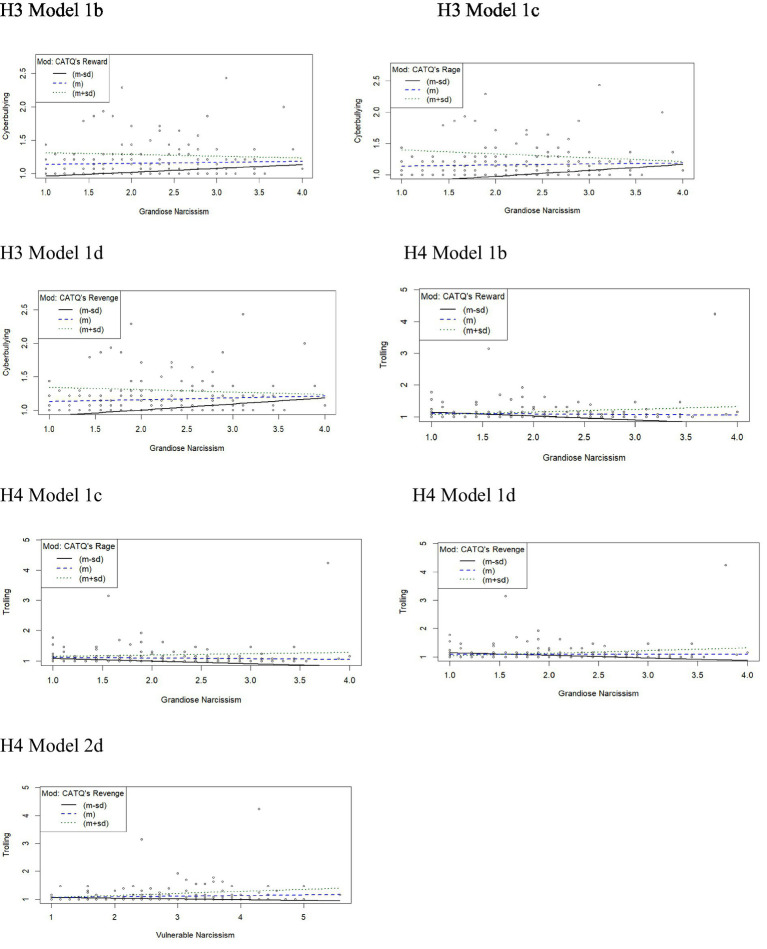
Graphics regression analysis for moderation: (H3 Model1) Relationship between grandiose narcissism and cyberbullying with each significant functional driver as moderator. (H4 Model1) Relationship between grandiose narcissism and trolling with each significant functional driver as moderator. (H4 Model 2) Relationship between vulnerable narcissism and trolling with significant functional driver as moderator.

Moderation analyses were performed to predict cyberbullying (H3) and trolling (H4) by considering the interaction between personality traits and functional drivers (as the moderator). For each hypothesis, two main models (Model 1 for grandiose and Model 2 for vulnerable narcissism), each of them including the four moderators, were analyzed, respectively. The results of the regression analyses are presented in Tables 3, 4 and simple slope plots were also illustrated (Figure 2) to detect the direction and magnitude of the significant interaction effects more effectively.

**Table 3:** Regression models for predicting cyberbullying by grandiose and vulnerable narcissism conditioned by levels of the four functional drivers.

Model	Predictor	β	SE	t	95% CI
H3 model 1	Constant	0.016	0.066	0.248	[−0.11, 0.15]
	DSHS – GE	0.027	0.060	0.401	[−0.10, 0.14]
	Recreation	0.484^***^	0.098	6.681	[0.31, 0.70]
	DSHS – GE * Recreation	−0.047	0.103	−0.742	[−0.25, 0.15]
	Constant	0.040	0.083	0.609	[−0.10, 0.23]
	DSHS - GE	0.051	0.076	0.753	[−0.11, 0.19]
	Reward	0.570^***^	0.211	5.515	[0.27, 1.09]
	DSHS – GE * Reward	−0.129^*^	0.149	−2.143	[−0.25, −0.01]
	Constant	0.070	0.082	1.061	[−0.08, 0.24]
	DSHS - GE	0.046	0.073	0.692	[−0.01, 0.19]
	Rage	0.767^***^	0.270	5.754	[0.21, 1.26]
	DSHS – GE * Rage	−0.243^***^	0.203	−3.534	[−0.38, −0.11]
	Constant	0.050	0.080	0.754	[−0.09, 0.22]
	DSHS - GE	0.082	0.075	1.213	[−0.07, 0.22]
	Revenge	0.647^***^	0.265	5.154	[0.21, 1.21]
	DSHS – GE *Revenge	−0.196^**^	0.205	−3.042	[−0.32, −0.07]
H3 model 2	Constant	0.025	0.072	0.369	[−0.13, 0.16]
	DSHS - ER	0.132	0.071	1.850	[0.00, 0.28]
	Recreation	0.451^***^	0.105	5.248	[0.23, 0.65]
	DSHS - ER * Recreation	−0.051	0.104	−0.992	[−0.15, 0.05]
	Constant	0.034	0.079	0.506	[−0.11, 0.20]
	DSHS - ER	0.196^**^	0.070	2.860	[0.06, 0.34]
	Reward	0.469^***^	0.183	4.033	[0.15, 0.86]
	DSHS - ER * Reward	−0.091	0.106	−1.349	[−0.28, 0.15]
	Constant	0.015	0.078	0.218	[−0.13, 0.18]
	DSHS - ER	0.253^***^	0.068	3.839	[0.12, 0.39]
	Rage	0.390^**^	0.246	3.008	[−0.01, 0.96]
	DSHS – ER * Rage	−0.060	0.162	−0.640	[−0.38, 0.24]
	Constant	−0.012	0.079	−0.183	[−0.14, 0.17]
	DSHS - ER	0.275^***^	0.070	4.185	[0.14, 0.41]
	Revenge	0.227	0.274	2.012	[−0.10, 0.99]
	DSHS – ER * Revenge	0.053	0.177	0.666	[−0.30, 0.48]

^*^p < 0.050; ^**^ *p* < 0.010; ^***^p < 0.000.

**Table 4:** Regression models for predicting trolling by grandiose and vulnerable narcissism conditioned by levels of the four functional drivers.

Model	Predictor	β	SE	t	95% CI
H4 model 1	Constant	−0.012	0.082	−0.179	[−0.18, 0.14]
	DSHS - GE	−0.057	0.087	−0.829	[−0.22, 0.12]
	Recreation	0.433^***^	0.172	5.840	[0.13, 0.75]
	DSHS – GE * Recreation	0.035	0.252	0.536	[−0.41, 0.52]
	Constant	−0.076	0.091	−1,260	[−0.21, 0.15]
	DSHS - GE	−0.048	0.067	−0.781	[−0.20, 0.07]
	Reward	0.207	0.225	2.184	[−0.11, 0.78]
	DSHS – GE * Reward	0.245^***^	0.280	4.435	[0.14, 0.35]
	Constant	−0.045	0.111	−0.740	[−0.23, 0.20]
	DSHS - GE	−0.048	0.087	−0.787	[−0.23, 0.10]
	Rage	0.329^**^	0.456	2.721	[0.45,1.29]
	DSHS – GE * Rage	0.153^*^	0.385	2.463	[0.03, 0.28]
	Constant	−0.056	0.076	−0.894	[−0.18, 0.13]
	DSHS - GE	0.001	0.069	0.010	[−0.17, 0.10]
	Revenge	0.121	0.238	1.027	[−0.22, 0.73]
	DSHS – GE * Revenge	0.218^***^	0.276	3.609	[0.10, 0.34]
H4 model 2	Constant	0.048	0.089	0.702	[−0.15, 0.20]
	DSHS - ER	−0.064	0.086	−0.867	[−0.22, 0.11]
	Recreation	0.546^***^	0.189	6.194	[0.20, 0.91]
	DSHS - ER * Recreation	−0.099	0.180	−1.887	[−0.29, 0.43]
	Constant	0.023	0.102	0.347	[−0.18, 0.23]
	DSHS - ER	−0.028	0.079	−0.414	[−0.17, 0.13]
	Reward	0.599^***^	0.312	5.287	[0.03, 1.21]
	DSHS - ER * Reward	−0.061	0.236	−0.925	[−0.42, 0.51]
	Constant	−0.022	0.104	−0.360	[−0.20, 0.21]
	DSHS - ER	0.049	0.072	0.799	[−0.09, 0.19]
	Rage	0.447^***^	0.394	3.732	[−0.21, 1.21]
	DSHS – ER * Rage	0.092	0.351	1.055	[−0.74, 0.62]
	Constant	−0.038	0.086	−0.594	[−0.18, 0.16]
	DSHS - ER	0.080	0.069	1.259	[−0.05, 0.23]
	Revenge	0.266^*^	0.336	2.444	[−0.28, 1.13]
	DSHS – ER * Revenge	0.167^*^	0.301	2.161	[0.01, 0.32]

^*^p < 0.050; ^**^ *p* < 0.010; ^***^p < 0.000.

The results related to H3 indicated that Model 1a was significant, F(3,198) = 19.15, *p* < 0.001, explained variance = 22.49%, although recreation did not significantly moderate the link between grandiose narcissism and cyberbullying, ΔR^2^ = 0.22%, F(1,198) = 0.55, *p* = 0.458, 95% CI [−0.16, 0.07]. Moreover, the three following variants of the model (Model 1b, 1c and 1d) were significant: Model 1b, F(3,198) = 16.29, *p* < 0.001, explained variance = 19.80%, Model 1c, F(3,198) = 16.70, *p* < 0.001, explained variance = 20.20%, and Model 1d, F(3,198) = 13.60, *p* < 0.001, explained variance = 17.09%. In addition, results indicated significant moderating effects of each functional driver on such relationship: Model 1b (reward), ΔR^2^ = 18.60%, F(1,198) = 4.59, *p* < 0.050; Model 1c (rage), ΔR^2^ = 5.03%, F(1,198) = 12.486, *p* < 0.001 and Model 1d (revenge), ΔR^2^ = 3.87%, F(1,198) = 9.25, *p* = 0.010.

When looking at the values of the conditional effect of the focal predictor at values of each moderator, results showed that the *p*-values associated with the conditional effects of both Model 1b and Model 1c (Figure 2) were higher and the established significance value and the 95% confidence interval included zero; in addition, a trend to a marginal significance was observed for revenge (i.e., *p* = 0.057) on the association between grandiose variant and cyberbullying behavior. As shown in Figure 2 (Model 1d), at a high level of such motivation (1 + SD), the effect size decreases, and the association between grandiose narcissism and cyberbullying is weakened. Consequently, the negative relationship between grandiose narcissism and cyberbullying becomes stronger for people with lower functional drivers.

When looking at Model 2 for H3 related to the associations between vulnerable narcissism and cyberbullying behavior, findings showed that the four models were significant, Model 2a, F(3,198) = 20.99, *p* < 0.001, explained variance = 24.10%, Model 2b, F(3,198) = 18.73, *p* < 0.001, explained variance = 22.10%, Model 2c, F(3,198) = 17.64, *p* < 0.001, explained variance = 21.10%, and Model 2d, F(3,198) = 15.92, *p* < 0.001, explained variance = 19.40%, although the four interaction effects were not significant, Model 2a (recreation), ΔR^2^ = 0.40%, F(1,198) = 0.98, *p* > 0.050; Model 2b (reward), ΔR^2^ = 0.70%, F(1,198) = 1.82, *p* > 0.050; Model 2c (rage), ΔR^2^ = 0.20%, F(1,198) = 0.410, *p* > 0.05, and Model 2d (revenge), ΔR^2^ = 0.20%, F(1,198) = 0.44, *p* > 0.05.

The results of Model 1 for H4 concerning the relationship between grandiose narcissism and trolling behavior showed that Model 1a was significant, F(3,198) = 15.32, *p* < 0.001, explained variance = 19.84%, although no significant interaction effect emerged, ΔR^2^ = 0.12%, F(1,198) = 0.29, *p* > 0.050. The other three models (Model 1b, 1c and 1d) were significant, Model 1b, F(3,198) = 32.06, *p* < 0.001, explained variance = 32.70%, Model 1c, F(3,198) = 34.76, *p* < 0.001, explained variance = 34.50%, and Model 1d, F(3,198) = 24.76, *p* < 0.001, explained variance = 27.28%, also indicating significant moderating effects of each functional driver on such relationship: Model 1b (reward), ΔR^2^ = 6.69%, F(1,198) = 19.25, *p* < 0.050; Model 1c (rage), ΔR^2^ = 2.01%, F(1,198) = 6.07, *p* < 0.001 and Model 1d (revenge), ΔR^2^ = 4.78%, F(1,198) = 13.02, *p* < 0.001.

The interaction plot showed that, as levels of reward and revenge increased (1 + SD), and their related effect size also increased (Figure 2, H4 Model 1b and 1d), the association between grandiose narcissism and trolling is strengthened. The positive association between grandiose narcissism and trolling was stronger for people with higher values in these two functional drivers. No significant *p* value was found for the conditional effect of the focal predictor at values of the moderator rage (Figure 2, H4 Model 1c).

Finally, significant findings emerged for H4 Models 2 related to the associations between vulnerable narcissism and trolling behavior: Model 2a, F(3,198) = 16.56, *p* < 0.001, explained variance = 20.10%, Model 2b, F(3,198) = 23.19, *p* < 0.001, explained variance = 26.00%, Model 2c, F(3,198) = 31.89, *p* < 0.001, explained variance = 32.60%, Model 2d, F(3,198) = 21.64, *p* < 0.001, explained variance = 24.70%. However, the interaction effects were not significant for the first three models, Model 1a (Recreation), ΔR^2^ = 1.40%, F(1,198) = 3.56, *p* > 0.050; Model 1b (Reward), ΔR^2^ = 0.30%, F(1,198) = 0.86, *p* > 0.050, and Model 1c (Rage), ΔR^2^ = 0.40%, F(1,198) = 1.11, *p* > 0.050. For the last one, Model 2d (Revenge), findings indicated a significant interaction effect, ΔR^2^ = 1.80%, F(1,198) = 4.67, *p* < 0.050. The graphic representation of the interaction effect showed that, as levels of revenge increased (1 + SD), and its related effect size also increased, the association between vulnerable narcissism and trolling was strengthened. The positive association between this variant of narcissism and trolling was stronger for people with higher values in this functional driver (Figure 2, H4 Model 2d).

The conclusion is warranted that (i) grandiose narcissists were less prone to cyberbullying, if they displayed marginal lower levels of revenge (controlled aversive dimension); (ii) grandiose narcissists were more prone to trolling when they manifested higher levels of reward (controlled appetitive dimension) and revenge; (iii) vulnerable narcissists were more prone to trolling when they manifested higher levels of revenge; (iv) no other significant effects of such variant of narcissism were observed on cyberbullying conditioned by functional driver (H3). Table 5 summarizes the results related to the hypothesized associations.

**Table 5:** General summary of the results.

Hypothesis	Results
H1 Model 1: Grandiose narcissism associated with cyberbullying	Not supported: positive association in bivariate correlations and no association in partial correlation
H1 Model 2: Vulnerable narcissism associated with cyberbullying	Supported: positive associations
H2 Model 1: Grandiose narcissism associated with trolling	Not supported: no significant associations
H2 Model 2: Vulnerable narcissism associated with trolling	Supported: positive associations
H3 Model 1: Cyberbullying was predicted by grandiose narcissism moderated by the controlled functional driver	Not supported: the association was weakened; a trend of significant interaction effects for revenge.
H3 Model 2: Cyberbullying was predicted by vulnerable narcissism moderated by a functional driver	Not supported: no significant interaction effects
H4 Model 1: Trolling was predicted by grandiose narcissism moderated by a controlled functional driver	Supported: the associations were strengthened; positive value of interaction effects for reward and revenge.
H4 Model 2: Trolling was predicted by vulnerable narcissism moderated by aversive functional driver	Supported: the associations were strengthened; positive value of interaction effect for revenge.

## 4 Discussion

This study aimed to examine the associations between the two variants of the narcissistic personality trait with cyberbullying and trolling behavior, and to shed light on the underlying motivations behind toxic online behaviors by disentangling individual differences in aggressive narcissistic actions. In general, the findings showed that the associations of the dual variant of narcissistic trait with the two forms of cyber-aggression were partially supported (i) and that the moderating role of functional drivers in individuals characterized by grandiose and vulnerable narcissism on toxic online behaviors was generally confirmed (ii).

However, a careful inspection of the findings revealed that the associations between grandiose and vulnerable narcissism with cyberbullying (H1 Model 1 and Model 2) and trolling behavior (H2 Model 1 and Model 2) were partially confirmed. In accordance with H1 Model 1, data showed mixed results, since the positive association of grandiose narcissism with cyberbullying emerged in bivariate correlation, but it was not confirmed in partial correlation, when the vulnerable variant was accounted for. Furthermore, H2 Model 1, dealing with the association between grandiose narcissism and trolling behavior, was not supported. After controlling the correlation of grandiose narcissism with two toxic online behaviors for vulnerable narcissism, the association between the constructs vanished. The lack of a significant association is in contrast with previous studies (Kjærvik and Bushman, 2021; Xu et al., 2024), although it is aligned with previous results (Rohmann et al., 2024; Goodboy and Martin, 2015), and with the assumption that the grandiose facet of narcissism may be considered the least consistently correlated with online offensive behaviors (Moor and Anderson, 2019). On the contrary, findings on the associations of vulnerable narcissism with cyberbullying and trolling confirmed H1 Model 2 and H2 Model 2, thus supporting the threatened egotism hypothesis linked to intrapersonal motive underlying the narcissistic actions. In fact, individuals characterized by vulnerable narcissism, having “thin skins” (fragile egos) tend to be sensitive to threats, criticism, and humiliation, and are more likely to respond with aggression online when they faced with negative evaluation. The significant association between the vulnerable facet and trolling aligns with similar studies (Bryce et al., 2023; Rohmann et al., 2024), and with the results obtained by Krizan and Johar (2015), who reported that vulnerable narcissism, rather than grandiose narcissism, was significantly related to trolling beyond the other dimension of narcissism.

An intriguing pattern of results also emerged when examining the effects of functional drivers acting as moderators in the associations between the dual nature of narcissism and toxic online behaviors. Indeed, a clear distinction between the two facets of narcissism emerged in connection with the two cyber-aggressive behaviors. The supposed relationship between grandiose narcissism and cyberbullying was not only confirmed (H3 Model 1), but results indicated an opposite direction of such association in relation to the expected prediction. Indeed, in contrast to our hypothesis, even if the trend was marginal, the higher negative values of revenge appeared to reduce the likelihood of grandiose narcissists to aggressively act in online interpersonal conflicts. Such results could be aligned with the SPIN hypothesis in connection with the underlying agentic factor of the grandiose narcissism (Miller et al., 2021) that includes extraversion, assertiveness, and leadership: individuals characterized by the grandiose narcissistic trait tend to be extravert, emotionally resilient, and socially charming at first impression (Fan et al., 2019). These characteristics lead them to develop good interpersonal relationships (McCain and Campbell, 2018; Carone et al., 2023). At the same time, to maintain higher levels of positive self-view, attention and admiration from acquaintances, other-oriented narcissists are less likely to participate in high-risk interpersonal attacks online, particularly when individuals know each other. One could also infer that the online situational context may lead to the acquaintances effect: the interaction with familiar people could lead grandiose narcissists to express aggressive acts through more communal means. The existence of the communal factor, considered as another content-specific variant of the agentic aspect of the grandiose narcissism is, therefore, supported (Gebauer et al., 2012). Indeed, being characterized by the need to be connected to others, have control over others and attain positions of authority, grandiose narcissists feed their unrealistically positive self-view of grandiosity, entitlement, and power by deliberately mitigating offensive behaviors and using intra- and interpersonal manipulative strategies, which involve warmth, agreeableness, and relatedness. Good interpersonal relationships may imply a development toward more cooperative communication and behaviors, although such strategies only serve as an ego-booster (Back et al., 2010). In this vein, such findings are in line with the investigation conducted by Du et al. (2022) showing that the agentic factor of the grandiose narcissism was associated with lower overall aggression and lower reactive aggression, and they also support the assumption that the grandiose facet of narcissism corresponds to the brightest trait or to the least dark trait (Rauthmann and Kolar, 2012).

Unexpectedly and contrary to the hypothesis, there is no evidence to suggest that functional drivers moderated the effects of vulnerable narcissism on cyberbullying. Therefore, H3 Model 2 was not supported. However, when looking at the direct relationship between functional drivers and cyberbullying, only a significant positive main effect emerged for the reward (the affective dimension underlying a positive emotional state). Such a result is consistent with the assumption that vulnerable narcissism is a variant characterized by a blend of neurotic and antagonistic features (Krizan and Herlache, 2018; Grapsas et al., 2020; Miller et al., 2017). Since vulnerable narcissists seek to experience positive emotional states by using planned strategies for their self-defence, they are more likely to be engaged in social conflict with others and to exhibit aggressive online behaviors.

A different picture emerged when looking at the relationships between grandiose narcissism and trolling behavior. The findings were in line with the expected hypothesis, H4 Model 1: the moderating effects of the functional drivers strengthened the associations between the two constructs due to the higher positive values of the motivational drivers. Unlike the context of cyberbullying, the situational context of trolling behavior leads to the anonymity effect, which can potentially increase the likelihood of acting aggressively. Such findings further corroborate the Status Pursuit hypothesis and previous findings (Kjærvik and Bushman, 2021; Furian and March, 2023), shedding light on the dynamics pertaining to narcissistic personality and influencing trolling behavior: narcissists with high levels of the grandiose component and with high levels of controlled functional driver tend to exhibit aggressive behavior for different purposes. Indeed, when they experience negative emotional states and are driven by intrapersonal motive (self/ego-oriented), they tend to act deliberately by lowering the status of their competitors or discrediting others’ success through revenge; when they crave for admiration and respect and are driven by interpersonal motive (other-oriented), they deliberately seek external/environmental validation of being recognized as superior by others (social status pursuit/reward). If grandiose narcissists are rejected by others or do not receive the respect they expect, they may experience negative emotions, like shame (Brummelman et al., 2018). In response to this aversive state, narcissists can turn the feeling of shame into anger (Thomaes et al., 2011), a phenomenon that was described as ‘narcissistic rage’ (Kohut, 1971), and manifest aggression. The identification of the dual behavioral mode of the grandiose narcissist trait can be mapped onto the combination of agentic and antagonistic characteristics, as previously shown (Krizan and Herlache, 2018; Grapsas et al., 2020; Miller et al., 2017).

Finally, the findings provided evidence for a significant association between vulnerable narcissism and trolling behavior moderated by high levels of revenge, thus supporting H4 Model 2 and the hypothesis of threatened egotism model. To defend their fragile ego and to exorcise uncomfortable emotions (e.g., shame, anxiety, and embarrassment) arising from perceived provocation, vulnerable narcissists, characterized by emotional vulnerability, tend to remove their negative emotional states by deliberately using a sort of calculated payback. The positive interaction effect of vulnerable narcissism by high level of controlled-aversive driver on trolling behavior has also demonstrated the importance of moving away from global assessments to more fine-grain ones, thus evaluating other underlying aspects that capture the interpersonal antagonistic feature of this variant (Du et al., 2022).

To sum up, the investigation has applied the R^4^ model (Runions et al., 2017) to explore how the two variants of the trait, interacting with motivational valence and cognitive processes, could modify the individual dispositions in engaging in cyberbullying and trolling behavior. The findings suggest that narcissists activate distinct sets of motivational valence, self-control processes and behavioral pathways in toxic online behaviors. Cyber-aggression acts as a function of interaction effects of each personality variant with different levels of functional drivers, and it also depends on the different situational contexts: when grandiose narcissists—characterized with low levels of affective-motivational drivers and self-regulatory process-interact with known individuals (cyberbullying), they are less likely to be engaged in aggressive behaviors; when they manifest high levels of functional drivers (reward and revenge) and interact with unknown individuals (trolling), they are more likely to act aggressively. Likewise, when vulnerable narcissists with high levels of revenge interact with unknown individuals, they tend to exhibit toxic online behavior.

The present study does not lack limitations, which must be addressed. Firstly, the sampling approach was convenience sampling, predominantly composed of undergraduate students, and women were over-represented. Therefore, these findings should be interpreted with caution due to the limitations associated with the use of a non-representative sample. In light of this, the generalizability of the findings remains uncertain, and any extrapolation beyond the studied population should be made carefully. However, it is important to note that the primary aim of this investigation, which is in line with a hypothesis-testing study, was to find out a relationship between the variables of interest. Thus, future researchers should prioritise more representative sampling strategies (e.g., randomised sampling strategies) to ensure adequate representation of key population subgroups in order to provide evidence for the generalizability of the discovery across different samples. Secondly, it is difficult to establish causal relationships due to cross-sectional analysis of the current study. To overcome such limitation, longitudinal design also using behavioral data should explore the causal relationships between the two variants of the personality traits and cyberbullying and trolling behaviors. To this purpose, it could be useful to connect language analysis with online behaviors—for example by collecting users’ lexical, morphological and semantic features characterized the online speech acts—to shed deeper insight into the relationships of linguistic correlates of the personality traits with toxic online behaviors. Indeed, the link of users’ personality-related language style information with their online behavioral features could not only improve the accuracy of personality prediction, by combining the data-driven-based approach focused on machine learning-based solutions with the theory-based approach focused on psychological theory of personality, but also design future and effective counterspeech interventions.

## Data Availability

The original contributions presented in the study are included in the article/supplementary material, further inquiries can be directed to the corresponding author.

## References

[ref1] Anderson C. A. Bushman B. J. (2002). Human aggression. Annu. Rev. Psychol. 53, 27–51. doi: 10.1146/annurev.psych.53.100901.135231 11752478

[ref2] Back M. D. Schmukle S. C. Egloff B. (2010). Why are narcissists so charming at first sight? Decoding the narcissism–popularity link at zero acquaintance. J. Pers. Soc. Psychol. 98, 132–145. doi: 10.1037/a0016338, PMID: 20053038

[ref3] Baumeister R. F. Smart L. Boden J. M. (1996). Relation of threatened egotism to violence and aggression: the dark side of high self-esteem. Psychol. Rev. 103, 5–33. doi: 10.1037/0033-295X.103.1.5, PMID: 8650299

[ref4] Bilewicz M. Tempska P. Leliwa G. Dowgiałło M. Tańska M. Urbaniak R.. (2021). Artificial intelligence against hate: intervention reducing verbal aggression in the social network environment. Aggress. Behav. 47, 260–266. doi: 10.1002/ab.21948, PMID: 33469962

[ref5] Bishop J. (2012). “The psychology of trolling and lurking. He role of defriending and gamification for increasing participation in online communities using seductive narratives” in Virtual community participation and motivation: Cross-disciplinary theories ed. Li H. (Hershey, Pennsylvania New York, USA: IGI Global), 160–176.

[ref6] Block J. H. Block J. (1980). “The role of ego-control and ego resiliency in the organization of behavior” in The Minnesota symposium on child psychology: Development of cognition, affect, and social relations. ed. Collins W. A. (Hillsdale, NJ: Lawrence Erlbaum), 39–101.

[ref7] Brummelman E. Nikolic M. Bögels S. M. (2018). What’s in a blush? Physiological blushing reveals narcissistic children’s social-evaluative concerns. Psychophysiology 55:e13201. doi: 10.1111/psyp.13201, PMID: 29876926

[ref8] Bryce C. J. C. Skvarc D. R. King R. M. Hyder S. (2023). Don’t set me off—grandiose and vulnerable dimensions of narcissism are associated with different forms of aggression: a multivariate regression analysis. Curr. Psychol. 42, 10177–10185. doi: 10.1007/s12144-021-02318-x

[ref9] Buchner A. Erdfelder E. Faul F. Lang A.-G. (2024). G*Power-Statistical Power Analyses for Mac and Windows. Available online at: https://www.psychologie.hhu.de/arbeitsgruppen/allgemeine-psychologie-und-arbeitspsychologie/gpower

[ref10] Bushman B. J. Anderson C. A. (2001). Is it time to pull the plug on hostile versus instrumental aggression dichotomy? Psychol. Rev. 108, 273–279. doi: 10.1037/0033-295X.108.1.273, PMID: 11212630

[ref11] Carone N. Benzi I. M. A. Parolin L. A. L. Fontana A. (2023). “I can't miss a thing”–the contribution of defense mechanisms, grandiose narcissism, and vulnerable narcissism to fear of missing out in emerging adulthood. Pers Individ Dif 214:112333. doi: 10.1016/j.paid.2023.112333

[ref12] Chen Y. Huo Y. Liu J. (2022). Impact of online anonymity on aggression in ostracized grandiose and vulnerable narcissists. Pers. Individ. Differ. 188:111448. doi: 10.1016/j.paid.2021.111448

[ref13] Du T. V. Miller J. D. Lynam D. R. (2022). The relation between narcissism and aggression: a meta-analysis. J. Pers. 90, 574–594. doi: 10.1111/jopy.12684, PMID: 34689345

[ref14] Fan C. Y. Chu X. W. Zhang M. Zhou Z. K. (2019). Are narcissists more likely to be involved in cyberbullying? Examining the mediating role of self-esteem. J. Interpers. Violence 34, 3127–3150. doi: 10.1177/0886260516666531, PMID: 27565705

[ref15] Furian L. March E. (2023). Trolling, the dark tetrad, and the four-facet spectrum of narcissism. Pers Individ Dif 208:112169. doi: 10.1016/j.paid.2023.112169

[ref16] Gebauer J. E. Sedikides C. Verplanken B. Maio G. R. (2012). Communal narcissism. J. Pers. Soc. Psychol. 103, 854–878. doi: 10.1037/a0029629, PMID: 22889074

[ref17] Goodboy A. K. Martin M. M. (2015). The personality profile of a cyberbully: examining the dark triad. Comput Human Behav 49, 1–4. doi: 10.1016/j.chb.2015.02.052

[ref18] Grapsas S. Brummelman E. Back M. D. Denissen J. J. A. (2020). The “why” and “how” of narcissism: a process model of narcissistic status pursuit. Perspect. Psychol. Sci. 15, 150–172. doi: 10.1177/1745691619873350, PMID: 31805811 PMC6970445

[ref19] Hangartner D. Gennaro G. Alasiri S. Bahrich N. Bornhoft A. Donnay K. (2021). Empathy-based counterspeech can reduce racist hate speech in a social media field experiment. Proc. Natl. Acad. Sci. USA 118, 1–3. doi: 10.1073/pnas.2116310118, PMID: 34873046 PMC8685915

[ref20] Hardaker C. (2010). Trolling in asynchronous computer-mediated communication: from user discussions to academic definitions. J. Polit. Res. Lang. Behav. Cult. 6, 215–242. doi: 10.1515/jplr.2010.011

[ref21] Hayes A. F. (2022). Introduction to mediation, moderation, and conditional process analysis (third). New York, NY, USA: The Guilford Press.

[ref22] Howard R. C. (2011). The quest for excitement: a missing link between personality disorder and violence? J. Forens. Psychiatry Psychol. 22, 692–705. doi: 10.1080/14789949.2011.617540

[ref23] Katz L. Harvey C. Baker I. S. Howard C. (2022). The dark side of humanity scale: a reconstruction of the dark tetrad constructs. Acta Psychol. 222:103461. doi: 10.1016/j.actpsy.2021.103461, PMID: 34902686

[ref24] Kjærvik S. L. Bushman B. J. (2021). The link between narcissism and aggression: a meta-analytic review. Psychol. Bull. 147, 477–503. doi: 10.1037/bul0000323, PMID: 34292012

[ref25] Kohut H. (1971). The analysis of the self: A systematic approach to the psychoanalytic treatment of narcissistic personality disorders. Chicago, IL: University of Chicago Press.

[ref26] Krizan Z. Herlache A. D. (2018). The narcissism spectrum model: a synthetic view of narcissistic personality. Personal. Soc. Psychol. Rev. 22, 3–31. doi: 10.1177/1088868316685018, PMID: 28132598

[ref27] Krizan Z. Johar O. (2015). Narcissistic rage revisited. J. Pers. Soc. Psychol. 108, 784–801. doi: 10.1037/pspp0000013, PMID: 25545840

[ref28] LimeSurvey GmbH. (2024). LimeSurvey. Available online at: https://www.limesurvey.org (Accessed January 2, 2025)

[ref29] McCain J. L. Campbell W. K. (2018). Narcissism and social media use: a meta-analytic review. Psychol. Pop. Media Cult. 7, 308–327. doi: 10.1037/ppm0000137

[ref30] Miller J. D. Back M. D. Lynam D. R. Wright A. G. (2021). Narcissism today: what we know and what we need to learn. Curr. Dir. Psychol. Sci. 30, 519–525. doi: 10.1177/09637214211044109

[ref31] Miller J. D. Hoffman B. J. Gaughan E. T. Gentile B. Maples J. Campbell W. K. (2011). Grandiose and vulnerable narcissism: a nomological network analysis. J. Pers. 79, 1013–1042. doi: 10.1111/j.1467-6494.2010.00711.x, PMID: 21204843

[ref32] Miller J. D. Lynam D. R. Hyatt C. S. Campbell W. K. (2017). Controversies in narcissism. Annu. Rev. Clin. Psychol. 13, 291–315. doi: 10.1146/annurev-clinpsy-032816-045244, PMID: 28301765

[ref33] Miller J. D. Maples J. (2011). “Trait personality models of narcissistic personality disorder, grandiose narcissism, and vulnerable narcissism” in The handbook of narcissism and narcissistic personality disorder: Theoretical approaches, empirical findings, and treatments eds. Keith Campbell W. Miller J. D. (Hoboken, New Jersey, USA: John Wiley & Sons, Inc.), 71–88.

[ref34] Moor L. Anderson J. R. (2019). A systematic literature review of the relationship between dark personality traits and antisocial online behaviors. Pers. Individ. Dif. 144, 40–55. doi: 10.1016/j.paid.2019.02.027

[ref35] Patrick C. J. Fowles D. C. Krueger R. F. (2009). Triarchic conceptualization of psychopathy: developmental origins of disinhibition, boldness, and meanness. Dev. Psychopathol. 21, 913–938. doi: 10.1017/S0954579409000492, PMID: 19583890

[ref36] Phan L. V. Rauthmann J. F. (2021). Personality computing: new frontiers in personality assessment. Soc. Personal. Psychol. Compass 15:e12624. doi: 10.1111/spc3.12624

[ref37] Quandt T. Klapproth J. Frischlich L. (2022). Dark social media participation and well-being. Curr. Opin. Psychol. 45:101284. doi: 10.1016/j.copsyc.2021.11.004, PMID: 35016088

[ref38] Rauthmann J. F. Kolar G. P. (2012). How “dark” are the dark triad traits? Examining the perceived darkness of narcissism, Machiavellianism, and psychopathy. Pers. Individ. Dif. 53, 884–889. doi: 10.1016/j.paid.2012.06.020

[ref39] Rohmann E. Marie Winkler S. Ozimek P. Bierhoff H. W. (2024). Are narcissists trolls? A cross-sectional study about aggression, trolling behavior, narcissism, and the moderating role of self-esteem. Telemat. Inform. 90:102122. doi: 10.1016/j.tele.2024.102122

[ref41] Runions K. C. (2013). Toward a conceptual model of motive and self-control in cyber-aggression: rage, revenge, reward, and recreation. J. Youth Adolesc. 42, 751–771. doi: 10.1007/s10964-013-9936-2, PMID: 23526207

[ref42] Runions K. C. Bak M. Shaw T. (2017). Disentangling functions of online aggression: the cyber-aggression typology questionnaire (CATQ). Aggress. Behav. 43, 74–84. doi: 10.1002/ab.21663, PMID: 27278715

[ref43] Ruocco A. C. Amirthavasagam S. Choi-Kain L. McMain S. F. (2013). Neural correlates of negative emotionality in borderline personality disorder: an activation-likelihood-estimation meta-analysis. Biol. Psychiatry 73, 153–160. doi: 10.1016/j.biopsych.2012.07.014, PMID: 22906520

[ref44] Thomaes S. Stegge H. Olthof T. Bushman B. J. Nezlek J. B. (2011). Turning shame inside-out: “humiliated fury” in young adolescents. Emotion 11, 786–793. doi: 10.1037/a0023403, PMID: 21604873

[ref45] Tokunaga R. S. (2010). Following you home from school: a critical review and synthesis of research on cyberbullying victimization. Comput. Human Behav. 26, 277–287. doi: 10.1016/j.chb.2009.11.014

[ref46] Trujillo A. Cresci S. (2023). One of many: assessing user-level effects of moderation interventions on r/The_Donald. ACM Int. Conf. Proc. Series 1, 55–64. doi: 10.1145/3578503.3583626

[ref47] Wink P. (1991). Two faces of narcissism. J. Pers. Soc. Psychol. 61, 590–597. doi: 10.1037/0022-3514.61.4.590, PMID: 1960651

[ref48] Xu W. Zhao B. Jin C. (2024). A meta-analysis of the relationship between personality traits and cyberbullying. Aggress. Violent Behav. 79:101992. doi: 10.1016/j.avb.2024.101992

[ref49] Zezulka L. Seigfried-Spellar K. (2016). Differentiating cyberbullies and internet trolls by personality characteristics and self-esteem. J. Digit. Forensic Secur. Law 11, 7–26. doi: 10.15394/jdfsl.2016.1415

